# Reconstructing sources location of visual color cortex by the task-irrelevant visual stimuli through machine learning decoding

**DOI:** 10.1016/j.heliyon.2022.e12287

**Published:** 2022-12-14

**Authors:** Yijia Wu, Yanni Zhang, Yanjing Mao, Kaiqiang Feng, Donglai Wei, Liang Song

**Affiliations:** a Academy for Engineering & Technology, Fudan University, Shang Hai, China; b Shanghai East-bund Institute on Networking Systems of AI, Shang Hai, China

**Keywords:** Computer, Machine learning, Decoding, Reconstructing, EEG, Visual, Color

## Abstract

Visual color sensing is generated by electrical discharges from endocranial neuronal sources that penetrate the skull and reach to the cerebral cortex. However, the space location of the source generated by this neural mechanism remains elusive. In this paper, we emulate the generation of visual color signal by task-irrelevant stimuli to activate brain neurons, where its consequences over the cerebral cortex is experimentally tracked. We first document the changes to brain color sensing using electroencephalography (EEG), and find that the sensing classification accuracy of primary visual cortex (V1) regions was positively correlated with the space correlation of visual evoked potential (VEP) power distribution under machine learning decoding. We then explore the decoded results to trace the brain activity neural source location of EEG inversion problem and assess its reconstructive possibility. We show that visual color EEG in V1 can reconstruct endocranial neuronal source location, through the machine learning decoding of channel location.

## 1 Introduction

In everyday circumstances, we are frequently accepted by a huge amount of visual color information interaction under complicated visual scenes. Our restricted perceptual resources need a recognition mechanism to prioritize the differences between different colors based on the subjective judgement of RGB, the basic elements of color in visual color information. For instance, when a task is designed that requires judgments on specific two different colors, the thinking mechanism invokes higher-level psychology and decision-making for analysis [1, 2, 3] to enhance the detectability and discriminability of visual areas that full within the task-relevant stimuli. Such task-relevant visual-spatial attention mechanism involves the triggering of a brain complex network regions [4], where the individual subjective judgments of the subjects are key for efficient recognition control [5, 6, 7]. Invasive studies using Functional Magnetic Resonance Imaging (fMRI) of the human brain’s responses to color have shown that these responses are similar to psychophysical measure [8]. fMRI cannot predict the outcome of behavioral adaptation [9], nor reveal relative time dynamics since it is restricted by the fairly slow time course of blood flow [10]. Therefore, fMRI cannot obtain high-accuracy classification of correlated events on a time course like EEG. Even with the higher spatial resolution of fMRI, the components that make up these signals cannot be analyzed efficiently. In addition, studies of human visual evoked potentials (VEPs) have shown that if photostimuli are observed under good spontaneous fixation, and its frequency and peak intensity remain exactly constant, it can occur approximately 12–30 s to at least 70 s after stimulus onset average steady state [11]. The intensity of the flash must be raised above the detection threshold for the human visual system to perceive the color [12]. This is evidence for the existence of color channels in the human visual system [13].

In parallel, research on stead-state visual evoked potentials (SSVEPs) have revealed the role of light stimulation in fixed-frequency orienting and EEG processing. To reflect a neural mechanism, the synchronization of specific brain rhythms within and between brain functional areas has been proposed by which at least one electrophysiological feature is being processed, whereby rhythms in the fixed EEG generated by visual stimuli seem to play a key role in real time decoding. In addition, it has been hypothesized that a SSVEP word spelling machine can achieve 90% typing accuracy [14], which raise and strengthen information pass by adjusting the frequency of flickering, and changes the sensitivity of visual cortex to afferent stimuli at the same time [15, 16]. Nevertheless, SSVEP cannot classify non-flickering colors [17] (flickering colors are essentially frequency decoding).

In this work, we explore the brain spatial nerve decoding mechanism using machine learning perceptual processing of color information in visual areas. We applied task-irrelevant stimulation on an arrayed electrode channel in the cerebral cortex to simulate the generation of visual color signals and tested if this activates stimuli cause V1 excitability fluctuations as a firing of locations generated within the endocranial nerve sources. In a first experiment, we show that neurons activation by task-irrelevant random color stimuli causes an significantly more inter-channel machine learning classification-decoding accuracy in V1 than other cortex as measured by simultaneous task-irrelevant random color stimulated multi-channel EEG. Next, we interrogate to what extent task-irrelevant random color stimulation activates neurons similarly causes visual sensing changes and V1 excitability through temporal characterization of VEP and ERP (experiment 2). In line with the machine learning classification-decoding results, we show that due to neuronal activation, firing, power, and excitability are also predominantly distributed across channels in the V1. For the first two experiments (experiment 1 and 2), we demonstrate the positive correlation between machine learning decoding and VEP power distribution on the spatial correlation through the EEG forward solution. In a final experiment (experiment 3), we reconstructed the source locations of endocranial neuronal firing activate by task-irrelevant random color stimulation using the results of machine learning classification decoding guided eLORETA and dipole localization method (DLM). Taken together, our results reveal an inverse EEG solution to the reconstruction of the location of perceptual neural sources guided by machine learning classification decoding within the visual ares. The revelation of the spatial distribution characteristics of electrophysiological signals of endocranial visual color information provides a certain contribution to provide a non-invasive method for neuroscience and therapeutic applications.

## 2 Materials & methods

### 2.1 Subjects

The subjects included 10 men and 10 women mean age 23.2 years with normal vision, and screened for colorblindness; All of them had no visual impairment and P300 experience. After experiment they received financial compensation ($30/h). All subjects in our study written informed consent and we received it. We confirming that informed consent was obtained from all subjects in our experiments. The experimental was approved by the Ethical Committee of the Graduate school, Fudan University. The experimental complied with the requirements stipulated by the Helsinki Treaty.

### 2.2 Task-irrelevant visual color stimuli

To collect the EEG sensory information of the subjects, the experiment used task-irrelevant stimuli. Task-irrelevant required subjects to remain relaxed and focused during the experiment. Compared with traditional task-relevant, task-irrelevant do not require subjects to perform decisions, judgments, reasoning, etc behaviors that will affect the final results during the experimental process according to prior design requirements. Such a design can prevent the EEG sensory information we collected during the experiment from being disturbed by other factors, ensure that the data is not contaminated, and maintain a high degree of purity.

**Figure 1:**
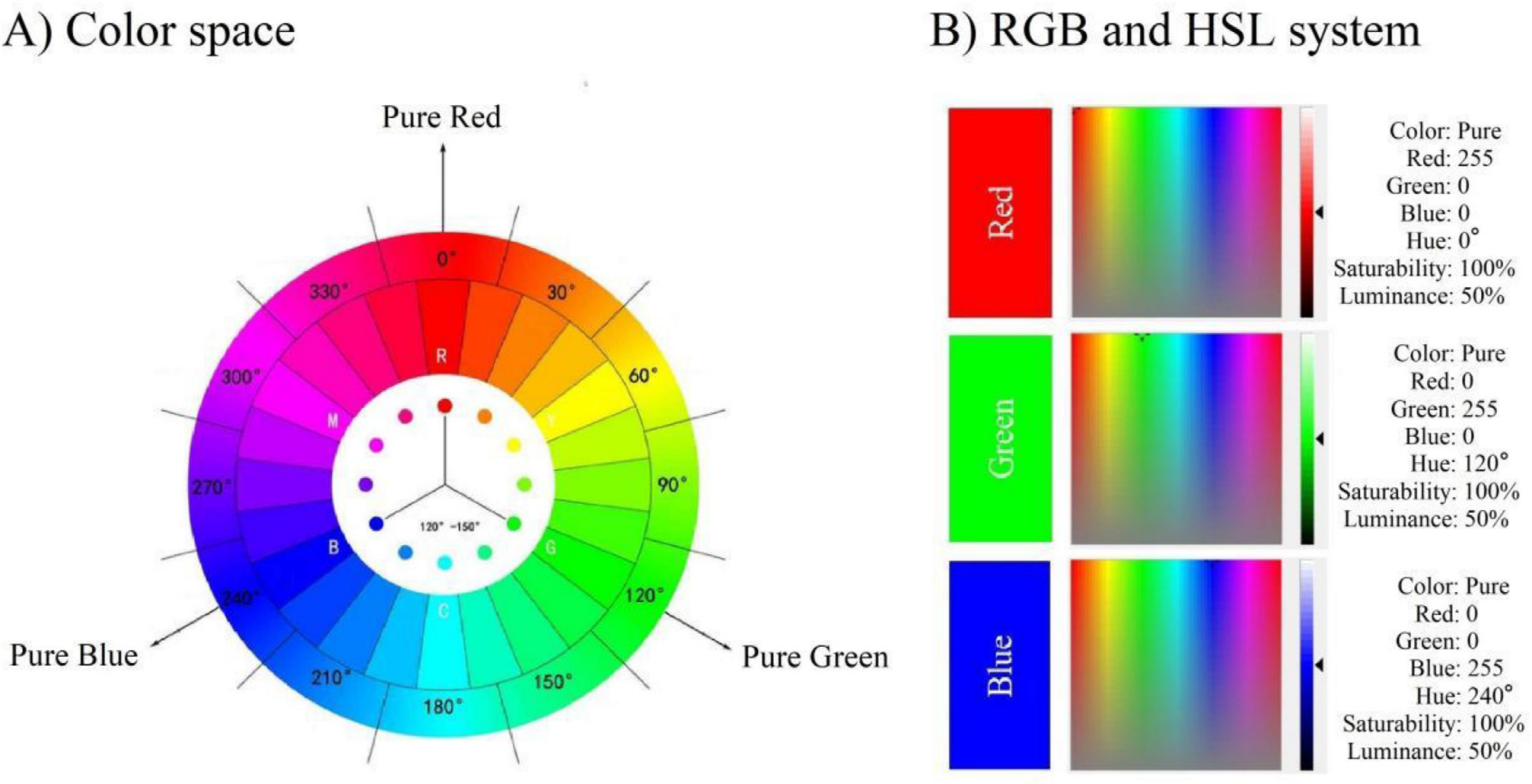
Visual color stimuli design. Figure 1-A) Color space. The Ostwald color system shows the location of experimentally selected pure color in the color space. The pure color is a high-contrast color at 120° to −150°. Choosing a high-contrast color can better evoke visual potential. In the color space, the Pure Red in 0° angle position, the Pure Green in 120° angle position, the Pure Blue in 240° angle position. Figure 1-B) RGB and HSL system. RGB system shows parameter values for color adjustment. HSL system shows that the Saturability and Luminance values have been fixed in addition to the color space location corresponding to Hue. Visual stimuli are not impacted by light. Pure Red Hue defined as 0°, Pure Green Hue defined as 120°, Pure Blue Hue defined as 240°. All Saturability were fixed as 100%, Luminance were fixed as 50%.

Stimulus targets are specially adjusted solid colors (pure red, pure green, and pure blue). We chose red, green, and blue because almost all colors in nature include these three elements. When the three color are displayed on the screen in the dark room, the luminance is uniformly adjusted to 3/5. Three color stimuli were individually appeared on a gray background (x = 0.32, y = 0.32, Y = 19.07 cd/m^2^). And the interior adjustment of the three colors adopts the Ostwald color space system (360°, 24 - color circle) (Figure 1A). In the RGB color space, pure red is defined as: 255,0,0; pure green is defined as: 0,255,0; pure blue is defined as: 0,0,255. By color convent converted to HEX hexadecimal code: #ff0000 (pure-red), #00ff00 (pure-green), #0000ff (pure-blue) (Figure 1B). In HSL color space, pure red is defined as: 0°; pure green is defined as: 120°; pure blue is defined as: 240°, saturability and luminance of the stimulus were fixed at 100% and 50% (Figure 1B). The aim of our experiment design in this way is (i) All color space parameter changes are only limited to RGB values, and the less the parameter changes, the less impact on the experiment; (ii) The impacts of inter-color saturability and luminance between different hues were excluded. We can estimate that the decoding results are unlikely to be affected by other factors.

### 2.3 Procedure

#### 2.3.1 EEG localization

Subjects seated in a dark room without any luminance, with their chin on chinrest (high 15 cm), kept 36 cm to the screen. Before starting the main experiment, all the channel locations on the EEG cap must be confirmed correct. In this work, we used the international general array scalp EEG distribution standard 59 channels system (from channel Fpz to O2) and the MNI coordinate system as leads [18]. The coordinates are centered on the CZ channel, first located in the centre of the sagittal line (using leather tape to measure), then adjust the equal spaces between left/right ears, nasal tip of the forehead and inion. The minimum error of location was controlled within 0.2 cm.

High level impedance creates more noise when recording EEG data. The noise will lead the accuracy of classification decoding deeply impact. In this work, before the recording of EEG data, a gelatinous medical coupling agent was applied to each channel. At the same time, we monitored the impedance of each electrode channel during the whole experiment and make sure the impedance of each channel must under 3 kΩ. During interaction, the synchronizer was used to reduce the error between the simulation time and recording time.

#### 2.3.2 Visual stimuli interaction

**Figure 2:**
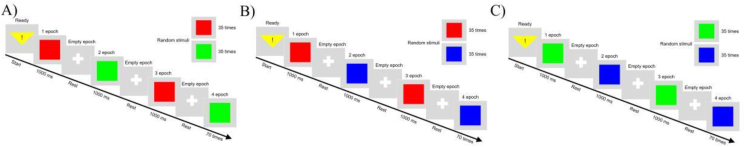
Visual color stimuli interaction paradigm. Figure 2-A) Red and Green (RG) team experimental paradigm. Figure 2-B) Red and Blue (RB) team experimental paradigm. Figure 3-C) Green and Blue (GB) team experimental paradigm. All subjects were accepted with EEG data collection while looking at the task-irrelevant random fast stimuli color for 1 s. The color have been adjusted by RGB and HSL systems. In each stimulus, the stimuli were randomly inserted into 210 epochs. Empty epochs during eye blinks were been deleted, and the rest epochs were generated 70 times per trail randomly. The figure depicts the experimental set-up and illustrates the stimulus interaction flow.

In this study we chose fast, random and active stimulation paradigm. This method can better restrain the subjects' self-consciousness during the EEG recording process. Fast stimuli can well evoke cells of visual rod neurons firing within a few milliseconds after stimulation [19]. Within a latency of 300 ms from external visual stimuli can be reflected by P300, but the color visual interaction latency is still not clear [20]. Therefore, each epoch interaction time windows were set for 1 s. During the process of visual interaction, it is very important to reduce the interference of other elements. Here, we using random stimuli combined with fast stimuli for the same time can solve this problem. Subjects were too late to respond within a short period, which ensuring that the EEG data only contained visual electrophysiological stimulation information. To record related random events, Event-Related Potential (ERP) was used to active stimuli. During supervised learning, it can be used to decode and classify colors. One epoch contains random 1 event and label in each interaction. The stimuli color of red, green, and blue are pairwise comparisons and were cross-validated for binary classification. The new team is defined: Red and Green – RG team, Red and Blue – RB team, Green and Blue – GB team. 3 trials in a team. In one trial, a random cross-interaction 35 times on average of one color. The two colors underwent a random cross-interacted 70 times on average in one trial. Each interaction was kept for 1 s. All 70 epochs would be generated in one trial. To tell the subjects to prepare for the start of the trial, a “yellow triangle” image was displayed on the screen before each trial. Then, on the centre of the screen a “white cross” image was displayed to encourage the subjects to concentrated and ready for the next interaction (Figure 2-A, B, C). Figure 2-A) shows RG team, Figure 2-B) shows RB team, and Figure 2-C) shows GB team. Resting time of each trial was adjusted according to the situation of subject’s. In this time, EEG data recording were stop. At last, all interaction time of two random colors with 3 trials in each team were 210,000 ms. One subject interaction time of trials with 3 teams were 630,000 ms.

#### 2.3.3 Data recordings and analysis (experiment 1)

Subjects performed EEG data collection in the Institute on Networking Systems of AI of the Academy for Engineering & Technology at the Fudan University (FDU), using the Start NeuSen W system. The experimental paradigm was created using Trigger Box.

First, we preprocessed to offset head movements. Then, raw data is sampled at 1000 Hz, and use notch filtering to remove power-frequency noise. Since power frequency noise range is over 50 Hz, and the EEG signals frequency effective range is under 30 Hz. The frequency information over 30 Hz was filter out by the basic finite impulse (FIR). We resampling frequency reduced from 1000 to 200 Hz to minimize the unrelated association sampling points. At last, to ensure the signal purity the eye movement artefacts was removed during the data processing. NSW 64 system was used to extract and saved the EEG data for every trial (from −200 to 1000 ms from before and after stimulus onset) with baseline mean removed.

MATLAB software was used to process EEG data. In the resting period during “white cross” was shown 210 epochs of empty were rejected (over 3 trials). Other included color information epochs divided into 210 epochs (in 3 trials) with 2 random color accounted for 105 in each team.

The decoding process was consisted by two parts. In first part, when the brain of subjects sensed the stimuli information after a short time, the Start NeuSen W system which was manufactured by Neuracle Technology (Changzhou) Co., Ltd started recording EEG data. In second part, the NSW 64 system transferred the EEG data to the classified decoding model.

**Figure 3:**
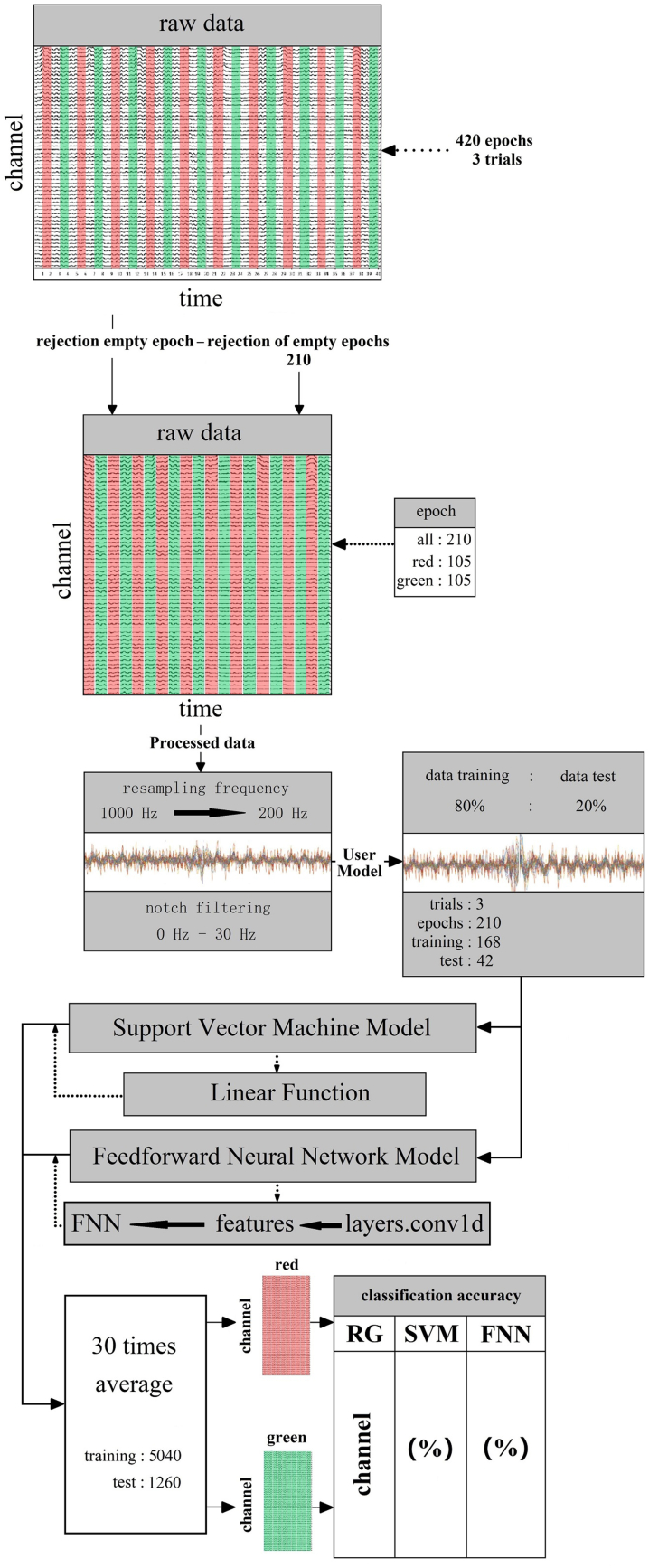
Machine learning decoding process. The classifier is mainly composed of support vector machines and neural networks. In raw data, each team contains 3 trails with 420 epochs. 210 empty epochs without visual color information are rejected. The remaining 210 epochs with color information are separated for 105 epochs with each color. Then preprocess the data. Resampling the frequency from 1000 Hz to 200 Hz and removed the noise of power frequency above 30 Hz through notch filtering. Set the ratio of preprocessed data training and test is 80%: 20% and the epochs for training are 168 for testing are 42 in 3 trails. Put the dataset into the main function of the SVM and neural network of FNN after cross-validations and take the 30 times average as the final result (training: 5040, testing: 1260).

Machine learning was used for decoding binary classification. We used the support vector machines (SVM) and the feedforward neural network (FNN) techniques based on supervised learning to train classifiers all 20 subjects 59 channels EEG data. We used the linear function as main functions of SVM for data analysis (After repeated attempts, kernel function and Gaussian function were not suitable in this study). In FNN, the neural network was mainly training, testing, and classifying data. The function layers. conv1d was used to extract features and input to FNN for classification (Figure 3). The ratio of the data training and data testing was constantly attempted and adjusted. Finally, the fit ratio were 8:2 (training datasets = 80%, testing datasets = 20%) (Figure 3). The iterations number were adjusted at the same time. When the iterations number exceeded 30, the system was overfitted after several adjustments. Finally, we used 30 iterations as the best fit resust for the system. The 30 iterations average value was used for classification decoding. In all 210 epochs, 168 are for training, 42 are for testing (Figure 3). All training datasets comprised 1260 epochs after 30 iterations (Figure 3). The final results of classification decoding were obtained through cross-validation (Figure 3).

The classification models can be run and construct under the platform environment of MATLAB 2019A and Python. The code of SVM and FNN can be found in the Science Data Bank (URL:https://www.scidb.cn/s/m63uM3). This code also fit other different configuration environments. All tables and figures data presented in this manuscript can be reproduced by the provided code.

#### 2.3.4 Dipole localization (experiment 3)

Among the tested algorithms, the eLORETA and DLM are able to provide the highest spatial accuracy. In addition, computer simulations show that the performance of the inverse solution is highly dependent on the number of active sources in the brain and the level of noise added to the signal. In this work, we carefully chosen the above two inverse models according to the signal type and noise level. We chose a real head model in this work. The real head model is obtained according to the actual structure of the human brain (according to MRI, X-CT and other imaging methods and anatomical knowledge of the human brain), which actually simulates the structure of the human brain, and the model is complex. The calculation and positioning accuracy of the real head model is very high, but the disadvantage is that there is no analytical solution, which can only be solved by numerical calculation. We decompose the EEG raw signal into multiple independent sources by channel, and locate each independent source separately according to the machine learning decoding results. According to Equation, the field distribution generated by the dipole at the origin of the coordinates can be obtained from formula (1). In formula (1), Ε(r,θ) is field strength distribution generated by the dipole at the origin of coordinates, $P_{0}$ is the magniitued of the electric dipole moment, $\hat{e_{r}}$ is the unit vector in the way of the line connecting the dipole to the observation point, $\hat{e_{\varphi }}$ is the unit vector perpendicular to the way, θ is the angle between $P_{0}\;and\;\hat{e_{r}}$.

(1) $$Ε\left(r,\theta \right)=\frac{P_{0}}{4\pi \varepsilon_{0}r^{3}}\left(2\;\cos\;\theta \hat{e_{r}}+\sin\;\theta \hat{e_{\varphi }}\right)$$

It is difficult to find the position of the dipole using formula (1): First, formula (1) assumes that the dipole is at the origin of the coordinates; Second, even if the coordinates are corrected, for we cannot know the position of the dipole and the connection of observation points, information such as $\theta ,\hat{e_{r}},and\;\hat{e_{\varphi }}$ cannot be obtained. But, by introducing the transfer matrix of formula (2), the electric field distribution formula (1) becomes formula (3).

(2) $$M=3\hat{e_{r}}\hat{e_{r}}-I$$

(3) $$E=\frac{1}{4\pi \varepsilon_{0}r^{3}}MP_{0}$$

We get the electric field generated by the electric dipole in space, and then determine the localization and size of the dipole through a series of formulas (2formulas (2) and (3)(3). The determination of the localization of a single electric dipole in space can be obtained by formula (4). In formula (4), $r_{0}$ is the coordinate of the dipole $P_{0}$.

(4) $$P_{0}=M^{-1}\left(rr_{0}\right)E\left(r\right)$$

Since the EEG usually uses 32 leads, generally only 32 field strengths can be obtained. Furthermore, due to the measurement error and the limitation of the data collection localization of some electrodes, the final determination of the localization of multiple dipoles is limited. Therefore, the general multi-dipole source locating is not easy to achieve.

## 3 Result

In this study, the three experiments designed are mainly used to answer the corresponding three questions: First, is the visual color sensing EEG data can be decoded by machine learning? Second, is the decoding result can be used for guided EEG space locating? Third, is the machine learning can be solved EEG inverse solution?

### 3.1 Experiment 1: machine learning decoding - EEG

Testing the hypothesis that signals originate at neurogenic location alter the excitability of V1 through power transfer, we randomly stimulated visual areas with an task-irrelevant (simulating “color”) and tracked activation effects through synchronous EEG recordings in the full brain. During the tracking process, we associate time and information one-to-one by marking Sync labels to corresponding stimulus events. Corresponding color stimuli were recorded using the corresponding label, and the same color was recorded using the same label. In the process of data recording, each event’s reflection and label recording are strictly corresponding, and the time difference system will automatically stop once it is reversed. After the experiment is completed, we only need to analyze whether data omission occurs during the experiment according to the actual number of labels. The transmission of nerve cell power was evaluated in terms of difference classification results among each channel, and compared under two classifiers, SVM and FNN, to explain the non-specificity of random stimulus of task-irrelevant caused by different colors. V1 responses were statistically compared with other cortex responses in the two classifiers, test including all electrode channels and a single time points within the first 500 ms time cut window locked to each visual interaction.

### 3.2 Nerve cell activation cause increased excitability of neural activity in V1

**Figure 4:**
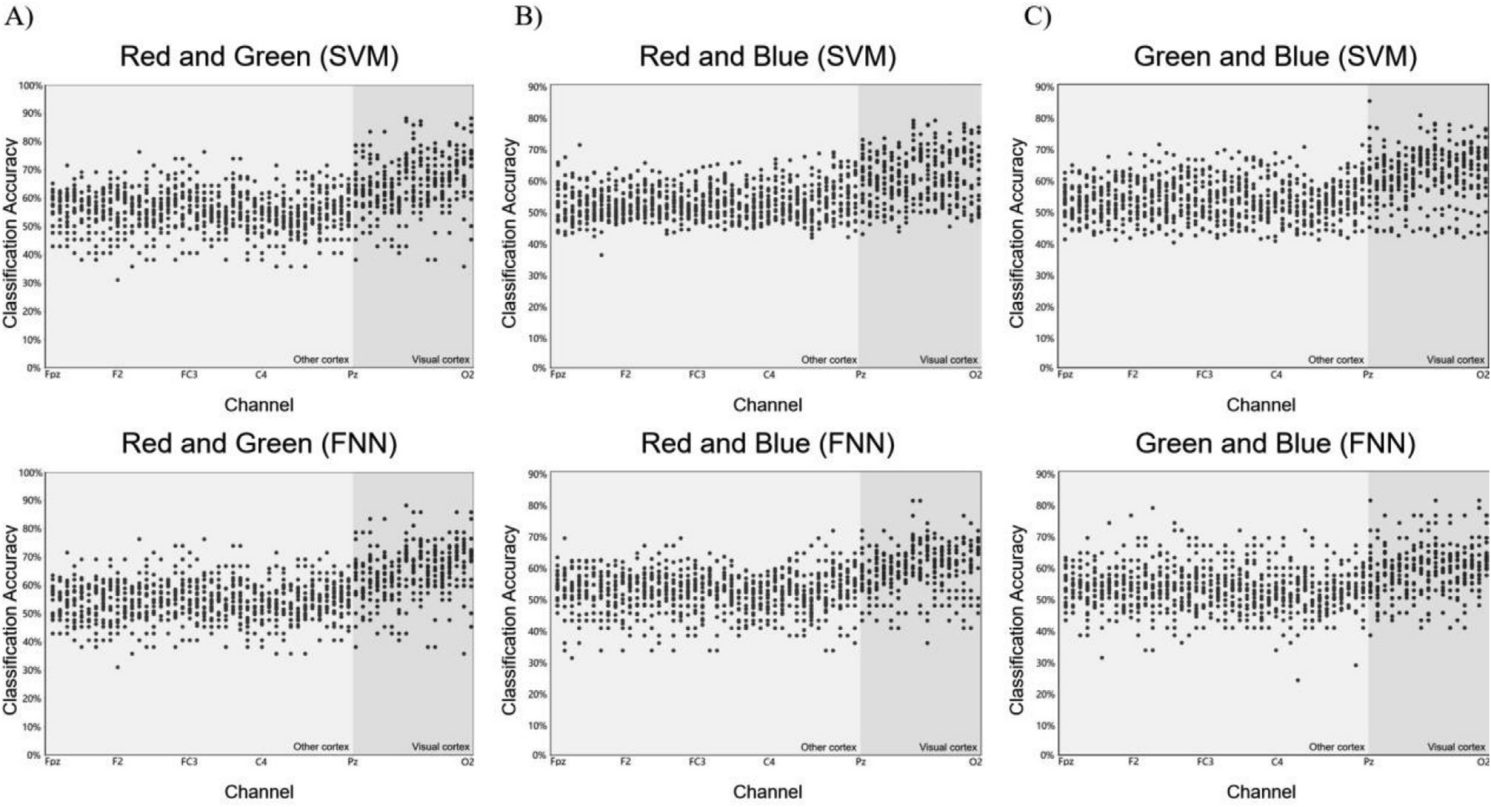
Basic results of classification decoding. Figure 4-A) shows the classification decoding results of RG team. Figure 4-B) shows the classification decoding results of RB team. Figure 4-C) shows the classification decoding results of GB team. Each team classification decoding results obtained under the two classifiers (SVM and FNN). The figure show all 20 subjects 59 channels classification results. It indicate that the channels with higher accuracy of classification obtained under both classifiers in each team almost in the V1 (dark gray background). The channel classification accuracy rate in the V1 is generally around 70%, and few can reach around 90%.

Globally, V1 neuronal activation improved classification accuracy over trials up to 300 ms and across multiple channels. Interestingly, these significant differences of classification accuracy in each channel were characterized by same topographical distributions suggesting that neuronal responses connect to same areas through specific stimuli. Channels with over 70% classification accuracy within the binary analysis were concentrated in the V1 (maximum on channel Pz, POz, Oz). Channels with a classification accuracy of 50% was limited to other cortex. Relevant to our hypothesis, we found that gradients of increased classification accuracy mainly involved V1 channels (Figure 4-A, B, C).

### 3.3 Using machine learning decoding results to analyze the neural-spatial feature mechanism of task-irrelevant random color stimulus activation

**Figure 5:**
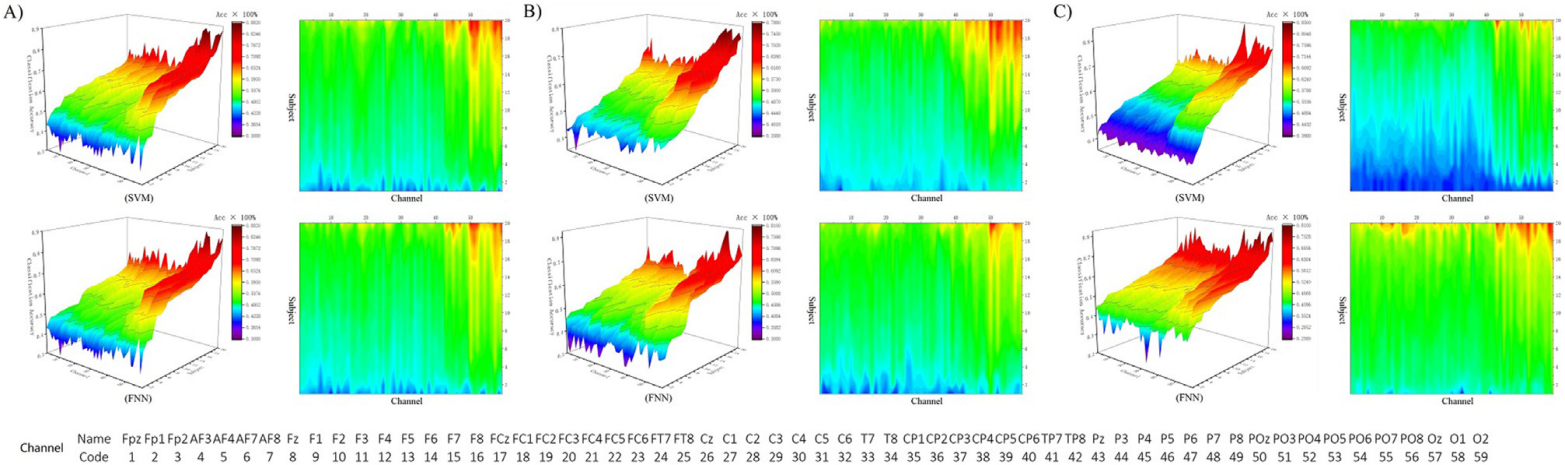
Decoding color information by 3D topographic map. Figure 5-A) The decoding results of team RG. Figure 5-B) The decoding results of team RB. Figure 5-C) The decoding results of team GB. In the left inset image, the topographic map, X-axis shows the channels form other cortex to V1, Y-axis shows the classification accuracy size change of subject’s channel, Z-axis shows the contour change of the classification accuracy. The space formed by the three sets of parameters shows the gradient of classification accuracy. In the right inset image, the 3D topographic map is Fourier transformed to obtain the mapping contour map. The counter map shows the distribution of decoding classification accuracy, which is in line with the result in Figure 4. This result verifies that the channel locations with higher classification accuracy obtained by machine learning decoding are concentrated in the V1. The bottom of Figure 5 provides standard ordering rules for channels and codes. A B C represents the results of team RG, RB, and GB under SVM and FNN respectively.

As further shown in Figure 5-A), Figure 5-B), and Figure 5-C) the average results obtained after 30 training and testing sessions on the EEG data constitute a three-dimensional data space on subjects and channels. The topography of the difference in the classification and decoding results points to the co-activation of the simulated V1, as express in the right image of Figure 5-A), Figure 5-B), and Figure 5-C) after converting the 3D topographic map into a contour map through Fourier transform, the channel with better decoding effect also concentrated in the V1 (Channel: Pz - O2; code: 43 - 59).

**Figure 6:**
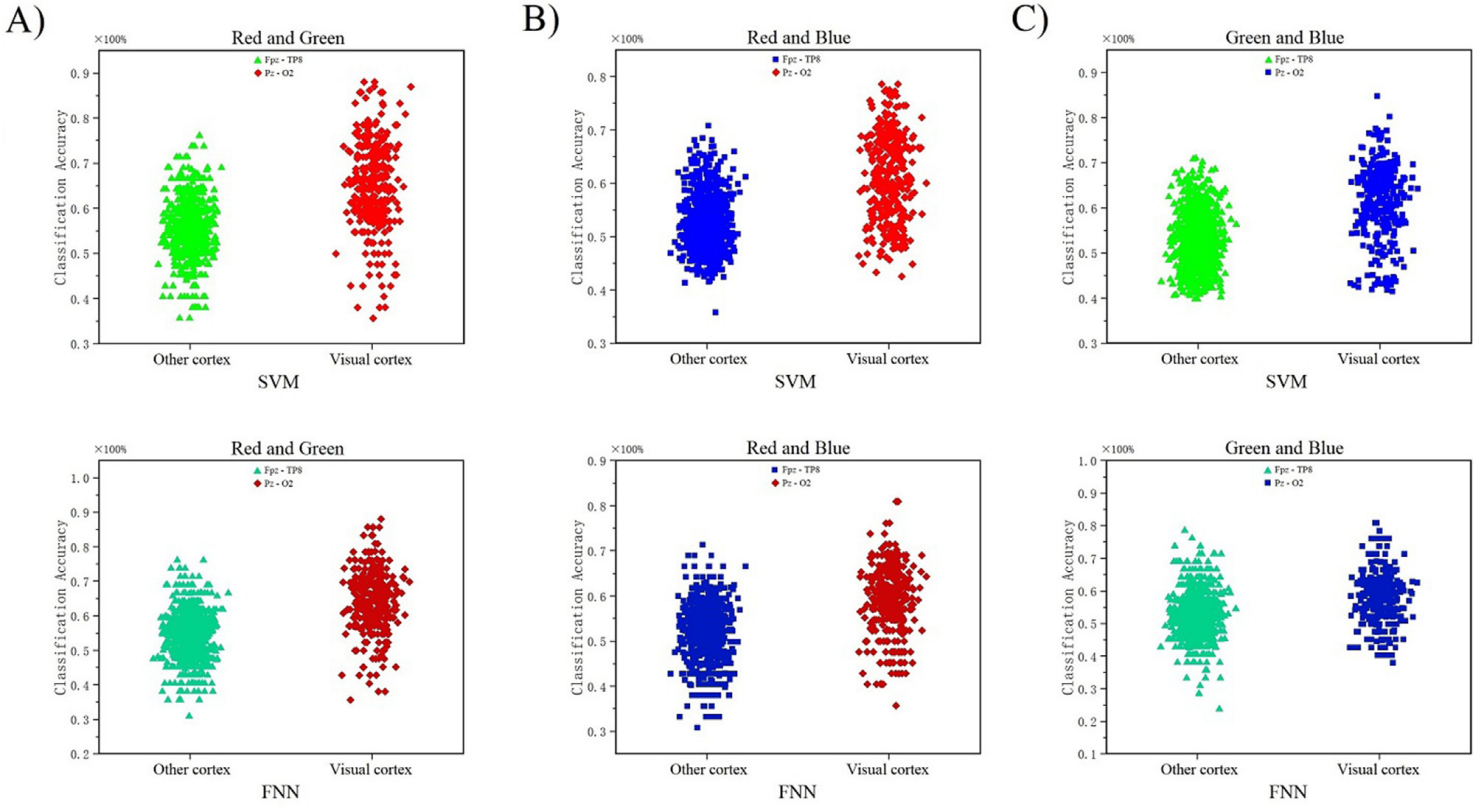
Comparing the results of other cortex channel decoding with V1 channel decoding. Figure 6-A), B), C) shows the channel decoding results under SVM and FNN of team RG, RB, and GB. In each team, the decoding results of SVM in line with FNN. All channels decoding results were divided into two teams (other cortex team and visual cortex team). By comparing the two cortex team, higher classification accuracy almost in the V1 team.

Figure 6-A), Figure 6-B), and Figure 6-C) shows the significant decoding difference between other cortex channel and primal visual cortex channel by statistical comparison analysis. In three teams, the decoding effect of V1 was more significant than other cortex under SVM and FNN model. From the scatter distribution in Figure 6-A), Figure 6-B), and Figure 6-C), after excluding the maximum and minimum values with a large degree of dispersion, the channel decoding classification accuracy of other cortex is relatively concentrated between 50% and 60%. It is obviously that the result is not ideal for the decoding of binary classification. The channel decoding classification accuracy of primal visual cortex is relatively concentrated between 60% and 80%. This results shows that when subjects receive two different color stimuli, the obtained EEG data are effectively distinguish by machine learning.

### 3.4 Performance validation of the classifier decoder

**Figure 7:**
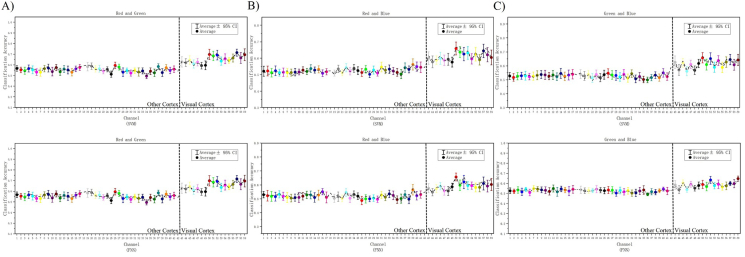
Comparing the 59 channel decoding results under SVM and FNN. Figure 7-A), B), C) shows the 59 channel decoding results of team RG, RB, and GB. In each team, the two curves represent the average line and median line of classification accuracy obtained of all 20 subjects under SVM and FNN. The fit of the two curves shows the difference in the results, and whether the decoding results are reliable. The dotted line divides the 59 channels into other cortex and visual cortex.

To test whether the machine learning can decode visual color information, we applied two classifiers (SVM and FNN) to decode EEG data of channels from Pz to O2 in V1. The machine learning algorithm training, testing and cross-validation on EEG data to identify difference between different color stimuli (see the details in “Methods”). The results shows that under both classifiers, there was a certain decoding rate at each electrode of interest within 300 ms of activate stimulus. Figure 7-A), Figure 7-B), and Figure 7-C)shows the average of 1180 channels of EEG data for 20 subjects (one subject contains 59 different channels, and the same channel contains data from 20 subjects) after 30 training and testing (The training and testing of the data are in single-channel units, and the cross-validation ratio: 80% for training set, 20% for testing set), and the results after statistics are performed in single-channel units (average ± 95%CI). We found that the decoding results obtained under the two classifiers are basically similar on the whole, and the overall curve formed by the mean values of each channel is basically fitted. In terms of the space channel location distribution, the gradients of the decoding results obtained by the two classifiers also have a clear span between the other cortex and the V1 (Figure 4, code: 42-43 marked by the dotted line).

**Figure 8:**
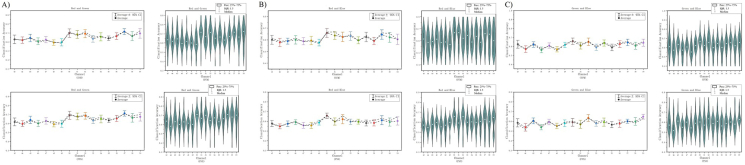
Decoding 17 channels of V1. Figure 8-A) Decoding results of RG team with SVM and FNN. Figure 8-B) Decoding results of RB team with SVM and FNN. Figure 8-C) Decoding results of GB team with SVM and FNN. In each team, left inset image shows the decoding results of 17 channels in V1 under SVM and FNN. The right inset image shows the classification accuracy statistics by box plot and violin plot of the V1 we are more interested in. The left inset image shows the results of average and median, and the right inset image show the results of probability statistics and distribution.

Figure 8-A), Figure 8-B), and Figure 8-C) shows the classification accuracy and decoding results of the 17 channels (channel: Pz-O2, code: 43–59) in the V1. We performed secondary statistics on the channels of the V1 and used box plots combined with violin plot analysis (The raw data is cut into quartiles, the box of the box plot is bounded by the upper quartile Q1 as the upper edge and the lower quartile Q3 as the lower edge, perc: 25%–75%, and the box retains 50% of the data, the median Q2 represents the more concentrated part of the dispersion). The results show that most of the Q2 > 60%, most of the nuclear density distribution probability over 60%, some good results can reach 70%–80%. At the same time, we also found an interesting phenomenon of space distribution: the three channels centered on Pz, POz, and Oz (code: 43, 50, 57) have the best decoding effect, and the location of these three channels are just right on the sagittal line.

**Figure 9:**
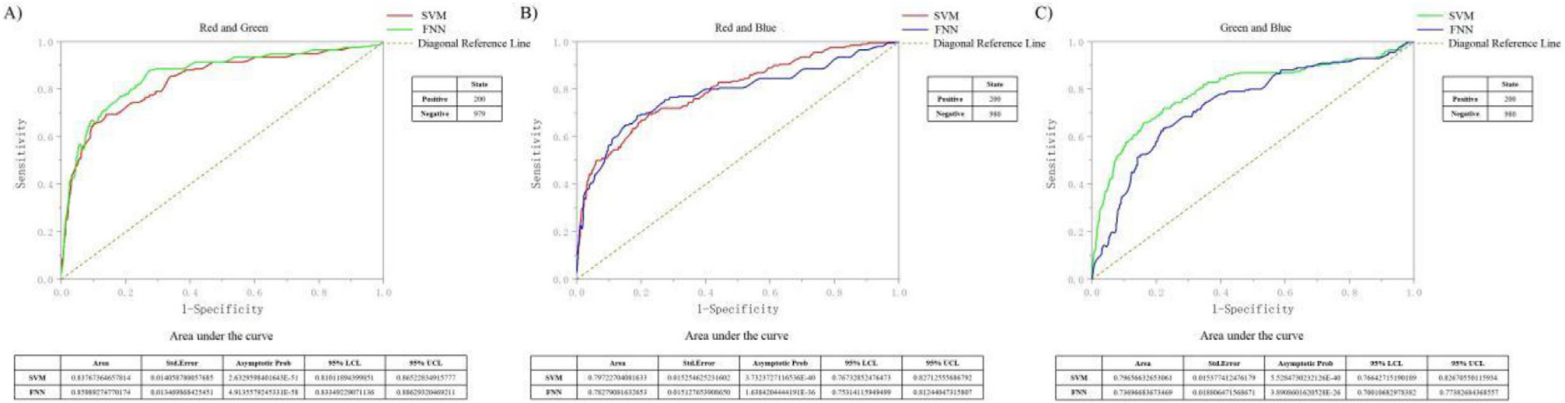
ROC curve and AUC results of Sub -1 to Sub - 20 on SVM and FNN. Figure 9-A) shows the ROC curve of RG team, line of red: SVM and line of green: FNN. Figure 9-B) shows the ROC curve of RB team, red line is SVM and blue line is FNN. Figure 9-C) shows the ROC curve of GB team, green line is SVM and blue line is FNN. The table below for each team represents the area under the curve. The table to the right for each team represents the state. The table under the figure shows the results of AUC, Std.Error, Asymptotic Prob, 95% LCL, 95% UCL. The right table shows the state of positive and negative.

Figure 9-A), Figure 9-B), and Figure 9-C) shows the 1180 data points formed by the 59 channel accuracy obtained on a classifier model for 20 subjects in each team. To test the performance of the two classifiers and perform Statistical Significance Testing, we split the 1180 data. The input data range of each team is 1180. Smaller test measurement values indicate more positive test. Larger test measurements indicate a biased test. The positive actual state is channel POz-O2. The state of positive is 200, Negative is 980 (all is 1180). Standard Error and Confidence Levels in 95%. Test Direction is Positive v.s. High. The results with diagonal reference line. In RG team, there are missing values in the input data (1 bad data, no effect on results). So the actual state of RG team is positive 200, negative 979. In RB and GB team, there is no bad data (missing values) - invalid values, and not used in calculations, no missing values. Finally, we got the AUC (Area Under the Curve) of RG was: AUC in SVM: 0.83767364657814; FNN: 0.85989274770174. Std.Error in SVM: 0.01-4058780057685; FNN: 0.013469868425451. Asymptotic Prob in SVM: 2.6329598401643E-51; FNN: 4.9135579245331E-58. 95% LCL in SVM: 0.81011894399851; FNN: 0.83349229071136. 95% UCL in SVM: 0.86522834915777; FNN: 0.88629320469211. In RB team the AUC are SVM: 0.79722704081633; FNN: 0.78279081632653. Std.Error are SVM: 0.015254625231602; FNN: 0.015127653908650. Asymptotic Prob are SVM: 3.7323727116536E-40; FNN: 1.6384204444191E-36. 95% LCL are SVM: 0.76732852476473; FNN: 0.75314115949499. 95% UCL are SVM: 0.82712555686792; FNN: 0.81244047315807. In GB team the AUC are SVM: 0.79656632653061; FNN: 0.73696683673469. Std.Error are SVM: 0.015377412476179; FNN: 0.018806471568671. Asymptotic Prob are SVM: 5.5284730232126E-40; FNN: 3.8908601620528E-26. 95% LCL are SVM: 0.76642715190189; FNN: 0.70010682978382. 95% UCL are SVM: 0.82670550115934; FNN: 0.77382684368557. The analysis results show that in the RG team, AUC of SVM: 0.84 > 0.5, FNN: 0.86 > 0.5, the results have certain accuracy. In the RG team, AUC of SVM: 0.80 > 0.5, FNN: 0.78 > 0.5, the results also have certain accuracy. In the GB team, AUC of SVM: 0.80 > 0.5, FNN: 0.74 > 0.5, the results also have certain accuracy. The sensitivity and 1-specificity of the two curves are biased towards the upper left of the diagonal reference line, respectively, which indicates that the two models we used have higher test accuracy. The basic fitting of the two curves once again shows that the results obtained by the model are similar and the model is relatively stable.

Taken together, these results suggest that: first, transient activation of neurons by task-irrelevant random stimuli leads to increased excitability of neural activity in V1; second, using machine learning can well decoded the electrophysiological neural space feature mechanism generated by visual color stimuli.

### 3.5 Experiment 2: The forward solution of EEG space location

**Figure 10:**
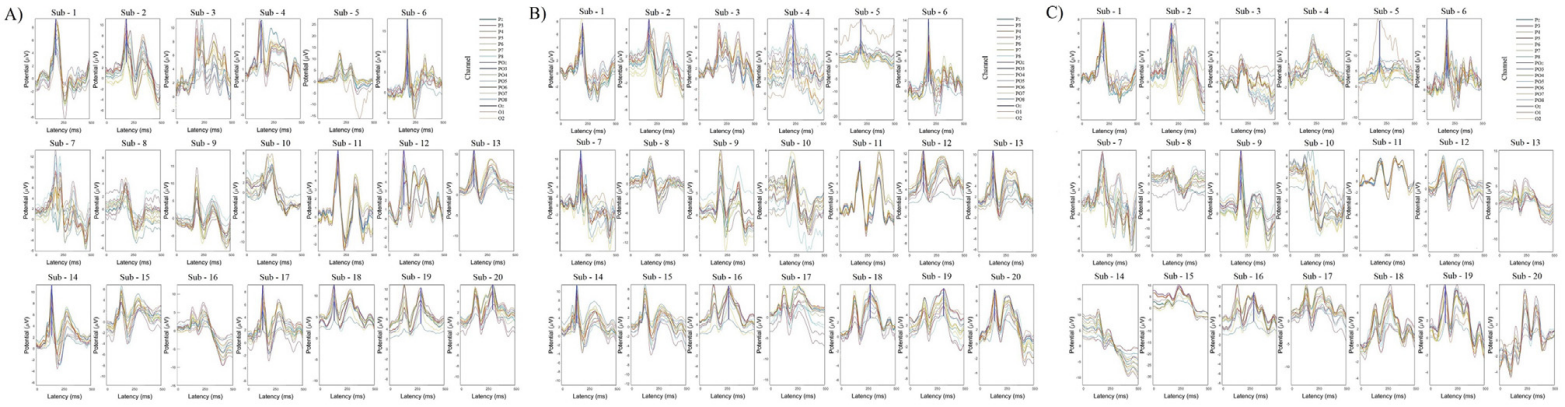
Cross-temporal decoding for visual color ERP. Figure 10-A) Cross-temporal decoding for visual color ERP of RG team. Figure 10-B) Cross-temporal decoding for visual color ERP of RB team. Figure 10-C) Cross-temporal decoding for visual color ERP of GB team. ERP subgraph show the time course of VEP for each subject in an epoch. The vertical axis express the value of EEG power potential (after one visual stimuli interaction), the horizontal axis express the latency of time courses (expressing that the time distance of latency from the stimulus occurrence). The curve shows the location of the first peak power after stimulation based on the P300 theory. Each curve shows the epoch in 3 trails for a channel.

Next, we sought to explain whether the pace distribution of channels decoded by machine learning is consistent with the location of VEP-evoked neuronal firing in cerebral cortex areas. If this were the case, the power peaks of cellular discharges should exhibit characteristics of ERP, in line with the mechanism theory of P300 in the temporal dimension. And the EEG experiment, we simulated fast stimulus signals through the Ostwald color space system, followed by task-irrelevant random stimuli in groups of three colors crossed in pairs. We reasoned that if the timing of power release elicited by VEP relative to evoked latency is correlated, we should be able to reveal the evoked periodicity of the stimulus by sampling epochs at various time points across trials. Stimulus activation followed by presentation with 17 different delays (one channel for each stimulus delay, here we only analyze 17 channels in the V1 with better decoding results, so a total of 17 delays), covering 500 ms time window (Figure 10-A, B, C). This design analyzes the downsampled 1000–200 Hz data between 0 and 30 Hz (the detailed description can be found in “Methods” section).

Figure 10-A), Figure 10-B), and Figure 10-C) shows all 20 subjects EEG data of channels in V1. Data visualization analysis intuitively shows that our previous speculation based on ERP and P300 is valid. In Figure 6-A), Figure 6-B), and Figure 6-C) represent the EEG changes of subjects within 500 ms after red, green, and blue stimuli. Each curve in the figure represents the average result of 105 epochs with one subject. We found that at least one peak of wave in power discharge occurred within the 500 ms time window after stimulus activation. This phenomenon suggests that the potential delay evoked by stimulus activation is consistent with ERP theory. At the same time, in the time course, the latency corresponding to the peak appeared before 300 ms, which was in line with the P300 theory.

**Figure 11:**
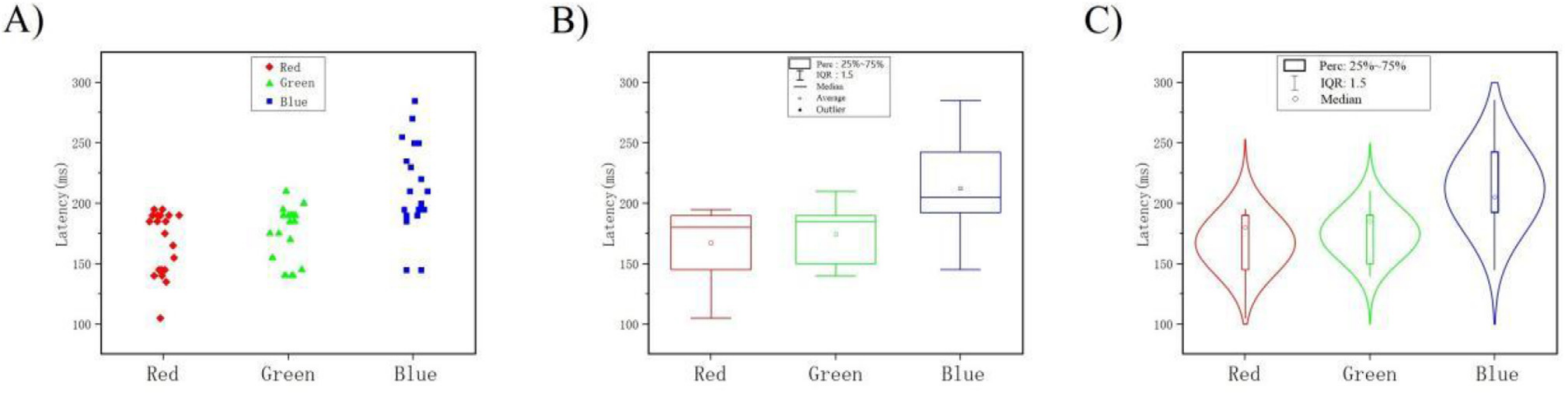
Decoding time courses. Figure 11-A) Continuous scatter distribution. The whole distribution of color time courses. Figure 11-B) Box plot distribution. The box plot show the centralized distribution position of the three datasets, median Q2 (Red: 180 ms; Green: 185 ms; Blue: 205 ms), upper quartile Q1 (Red: 190 ms; Green: 190 ms; Blue: 235 ms), lower quartile Q3 (Red: 145 ms; Green: 155 ms; Blue: 195 ms), Avg (Red: 167.25 ms; Green: 174.75 ms; Blue: 212.5 ms) of each box. Box perc range: 25%–75%, IQR: 1.5. Figure 11-C) Violin plot distribution. The violin plot show the Kernel density distribution. The lateral kernel density is normally distributed, and most of them in line with the box plot and scatter distribution.

Figure 11-A), Figure 11-B), and Figure 11-C) shows our statistics on the ERP results. First, we classified the EEG latency generated by the three color stimuli into teams (red team, green team, blue team), and analyzed the data distribution of each team through scatter plots (Figure 11-A). In Figure 11-A), there are differences in the overall distribution of the incubation period of three color teams. Most of the data points are distributed relatively convergent, and few of the data has a large dispersion. In order to better reflect the overall distribution of the data, we further statistical analysis. We performed a statistical analysis of three color teams latency using box plots. The raw date for each team is cut into quartile, and the upper quartile Q1 and lower quartile Q3 are used as edges to form the box. 50% of the total data volume is retained in the box (perc: 25%–75%). After sorting, the red team Q1 = 190 ms; Q3 = 145 ms; the green team Q1 = 190 ms; Q3 = 155 ms; the blue team Q1 = 235 ms; Q3 = 195 ms; we found that after removing the more discrete values, 50% of the data in the valid box reflects the median Q2 of each team is red Q2 = 180 ms; green Q2 = 185 ms; blue Q2 = 205 ms, while the mean of each team is red Avg = 167.25 ms; green Avg = 174.75 ms; blue Avg = 212.5 ms (Figure 11-B). Figure 11-C) shows the statistical distribution of kernel densities and probabilities at time points. The results predicted the distribution range of the incubation period of the tricolor and the location where it might be concentrated. We found that red and green had shorter and similar latency, and blue had longer latency.

This result suggests that it is feasible to decode color in the temporal dimension using ERP and P300 theoretical mechanisms combined with machine learning. At the same time, these results also show that when the brain perceives color stimuli, the red color is evoked the shortest time, followed by the green color and the blue color. It is also explains why the red color draws the fastest visual attention of human, and then followed by the green color and the blue color. This may also explain why the red color is used to alert others and indicate danger [21].

**Figure 12:**
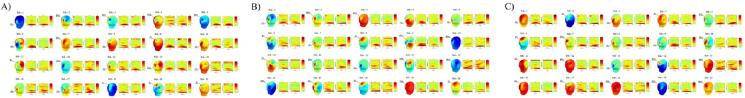
Comparing the machine learning decoding with VEP power distribution on time frequency. Figure 12-A) The comparing result of RG team. Figure 12-B) The comparing result of RB team. Figure 12-C) The comparing result of GB team. In each subjects, the left inset 3D-plot image shows two results. The channel shows machine learning decoding results, and the color shows VEP power distribution results. These two results in the same space location (MNI space coordinates). The right inset time frequency image shows decoding results of machine learning and power discharge on time courses.

Take into account the experimental design is feasible based on the theoretical mechanism of ERP and P300, we overlap the EEG dataset result of machine learning decoding and VEP power distribution. Partial overlap was necessary to analyze whether there is a positive correlation between the decoding results and the power distribution on the 59-channel standard array cortical EEG (based on the MNI space coordinates). Figure 12-A), Figure 12-B), and Figure 12-C) shows the correspondence between the epoch machine learning decoding results and the VEP power distribution for each subject. Through the channel analysis of the V1 on the extracranial 3D-plot, In line with the machine learning decoding results, the neural cell activation caused by visual color stimuli can best explain the three channels of Pz, POz, and Oz (Figure 12-A, B, C, left inset 3D-plot The channel indicates the machine learning decoding result). The statistical results and all the results express that in occipital lobe prat of the V1, classification accuracy of the three channels longitudinally arranged on the sagittal line with Pz, POz, Oz as the center is higher (Figure 8, left inset image Acc Avg: 70%–80%. Figure 12-A), Figure 12-B), and Figure 12-C), left inset image 3D-polt channel location), and then subjected to extracranial cortex EEG localization. As shown in Figure 12-A), Figure 12-B), and Figure 12-C) (left inset scalp color topography and right time frequency map), the three-dimensional data map of VEP time frequency after fast Fourier transform significantly explains the power release process of neural activation over the time course, The range is from 0 to 30 Hz (above 30 Hz is power frequency noise, which has been removed by sag filtering in data preprocessing) and a window sampling rate 200 Hz (invalid correlation between data points formed sampling rate 1000 Hz is high, so resampling from the original 1000 Hz–200 Hz). For each window, the time course from 0 to 400 ms was also significant. Considering that the result of VEP power distribution and machine learning decoding need to be compared in the same space, we then tested whether the time frequency of a single subject is mapped consistently on the 3D-plot. We found that the firing volume of neuronal activation within 300 ms is in line with the theoretical mechanism of ERP and P300 (Figure 10-A, B, C), the power size of the time frequency in the window is in line with the 3D-plot topographical distribution (Figure 12-A, B, C, left inset 3D-plot color topographic map, the color represents the value of the released power).

Finally, as for experiment 2, we analyze and compare whether the machine learning decoding results are in line with the VEP power distribution results. Our aim was to verify once again whether machine learning decoding could be attributed to the ERP like response produced by VEP activation. For this reason, we placed the machine learning decoding results and the VEP power distribution results overlapping in the same 3D-plot space (Figure 12-A, B, C, left inset). We compared the space distribution of the two results (Figure 12-A, B, C, left inset). We found that the machine learning decoding results are mainly concentrated on the three channels of Pz, POz, and Oz (the channel locations marked in the left inset of Figure 12-A, B, C). The space location of VEP power release is also in the frontal lobe of the V1 (the darker colored topographic map in the left inset of Figure 12-A, B, C). By comparison, it can be found that the result of power distribution and decoding appearing in the 300 ms latency are basically consistent in the space location distribution.

In summary, these results demonstrate that machine learning can decode EEG evoked by visual color sensing, and is basically consistent with the results obtained by the traditional VEP method in the temporal and space dimensions, and both are positively correlated. This result also suggests the possibility of using machine learning as another way to decode the space location dimension of EEG.

### 3.6 Experiment 3: The inversion solution of EEG space location

**Figure 13:**
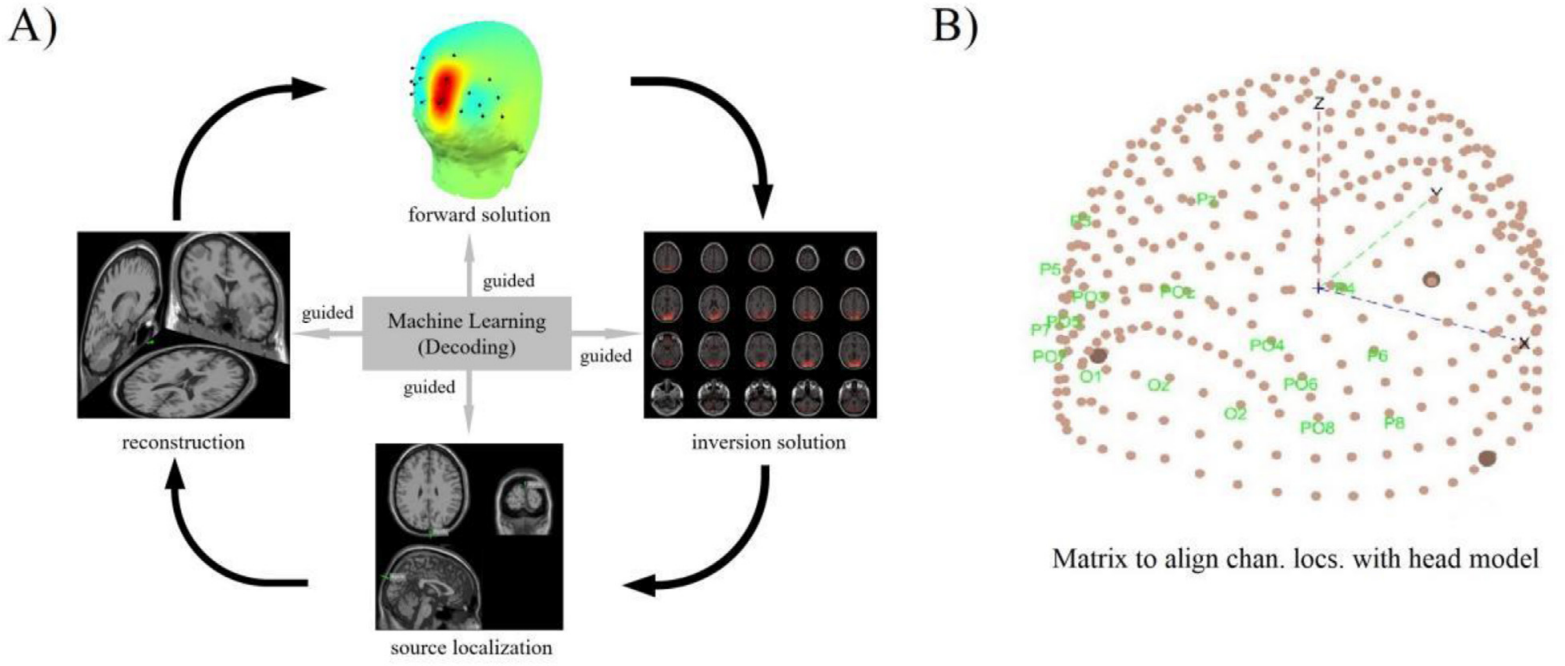
EEG traceability positioning solution. Figure 13-A) Using machine learning to solve EEG traceability positioning solution. Figure 13-B) Head model. Establishment of space coordinates from extracranial to endocranial.

The inverse solution of EEG spatial localization, also known as the EEG inverse problem, can be summed up as: measuring the potential signal according to the head table, inverting and estimating the position, direction and intensity information of the neural activity source in the brain (Figure 13-A). Essentially, the EEG inverse problem should be a nonlinear optimization problem. Considering the complexity of the calculation, the approximate reduction is the linear problem Y = AX, where Y is the point recorded by the head surface electrode, and X is the space to be performed. The localized source information vector, A called the transfer (gain) matrix, is the solution to the EEG positive problem and can be obtained by constructing a suitable head model etc (Figure 13-B). Here, we use the DLM for EEG traceability and localization. DLM is an effective non-invasive analysis method. It uses electric field theory and computer technology to calculate the hypothetical source of this electrical activity by brain based on the distribution of EEG scalp. DLM advantages are that there are few parameters, the software is easy to operate, and there is a large parameter space. The mathematical problems generated are linear, but the positioning cannot be expressed quantitatively. Therefore, we use the quantitative results (classification accuracy) obtained by machine learning decoding combined with DLM to trace the origin of EEG space.

### 3.7 Machine learning-guided EEG source localization (e-Loreta)

fMRI-guided EEG source location. In the resolution of spatial, EEG haven’t had the incomparable advantage of fMRI. However, in some cognitive tasks, the activated brain areas may change on a small time scale. If this change exceeds the observation ability of fMRI, the parameters in the two dimensions on time and space cannot be one-to-one correspondence. Furthermore, fMRI is essentially another way of measuring EEG signals. The collection of data needs to be completed on two different experimental platforms, and the analysis of the results needs to be processed later in the time course to achieve mutual agreement. Therefore, considering these complexities, we propose in this experiment the use of machine learning to guide EEG source localization. The main advantage of using machine learning to guide EEG source localization are: data acquisition only needs to be completed once. All experiments were done on the same experimental platform. Second, the results obtained by machine learning have a very high resolution in time resolution, which can be down to milliseconds like EEG. At this point it makes up for the deficiencies of fMRI.

We performed source localization experiments on the collected EEG data of 20 subjects and used subject-specific source localization for EEG analysis. The aim is to trace the location of the endocranial signal source using the channel with high classification accuracy obtained by machine learning in combination with the power feature distribution.

**Figure 14:**
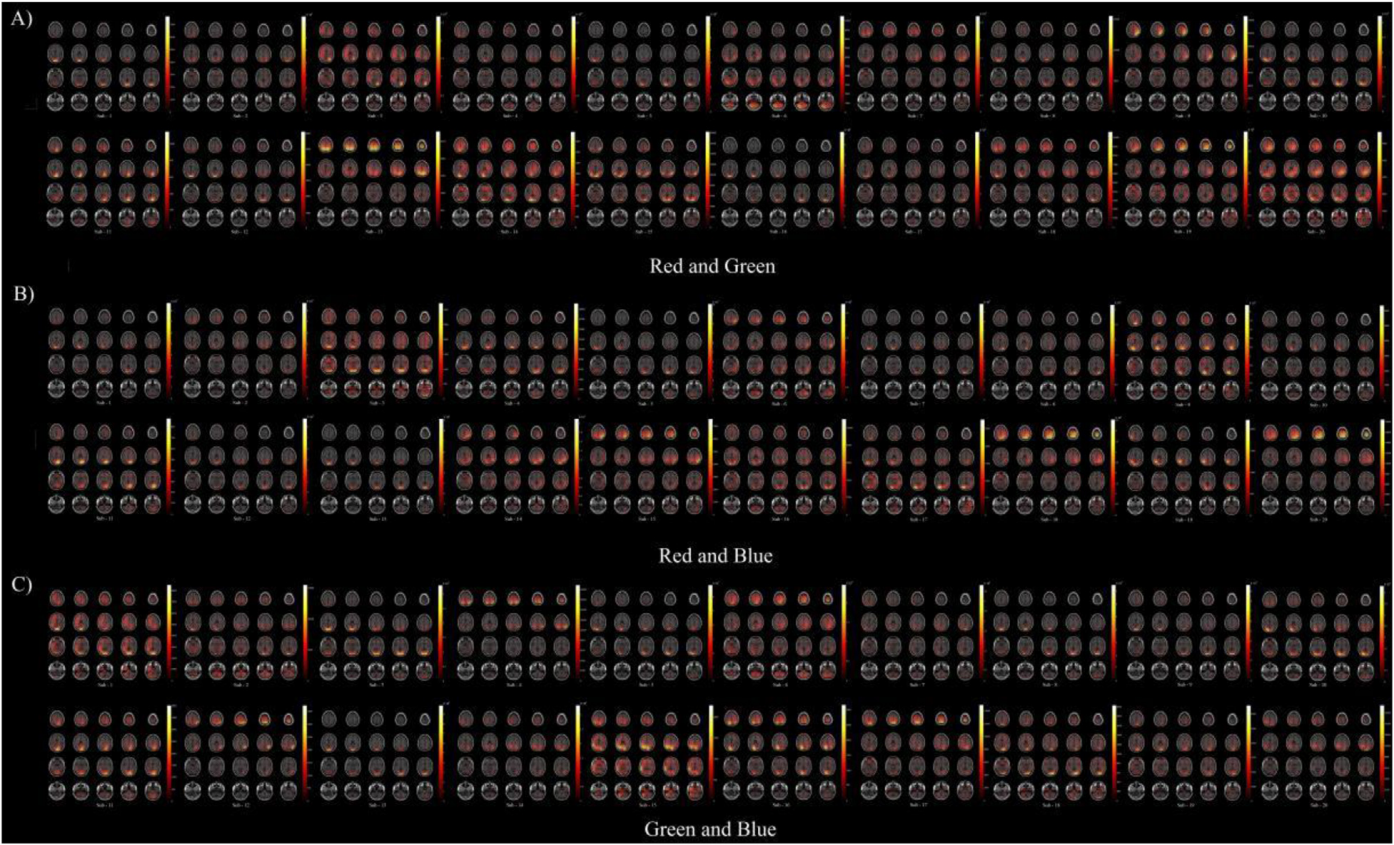
eLORETA reverse positioning. Figure 14-A) The result of team RG reverse positioning. Figure 14-B) The result of team RB reverse positioning. Figure 14-C) The result of team GB reverse positioning. Each subjects contain 4 MRI layers from the bottom to the top of the skull. One layer were cut into 4 pieces. The luminance shows the high power which have strong response and neural activity for visual color stimuli. The results of these inverse simulations are all from Figure 12 left inset 3D-plot image.

Figure 14-A), Figure 14-B), and Figure 14-C) shows the classification results of machine learning in teams red-green, red-blue, and green-blue combined with VEP power distribution using eLORETA traceability localization results: MRI heat map shows 20 slices from the top of the skull to the base of the skull scan results. We found that there was a large energetic response to the location of the V1 after visual color stimuli, and the correspondence was evident in the endocranial occipital lobe. The brighter the color of the area, the greater the power, the more obvious the feature, and the greater the stimulus feedback.

The MRI slice located in the second row shows that the EEG signal source produced the highest current source density of all stimuli. The location of this tomogram did not differ from the location of the channel with the highest machine learning classification accuracy. This result provides neural activity in endocranial direct decoding and corroborates oblique measures obtained by machine learning classification, suggesting that regions of machine learning decoding guided eLORETA retrospective localization play an important role in reconstruction.

### 3.8 Machine learning-guided EEG space reconstruction

**Figure 15:**
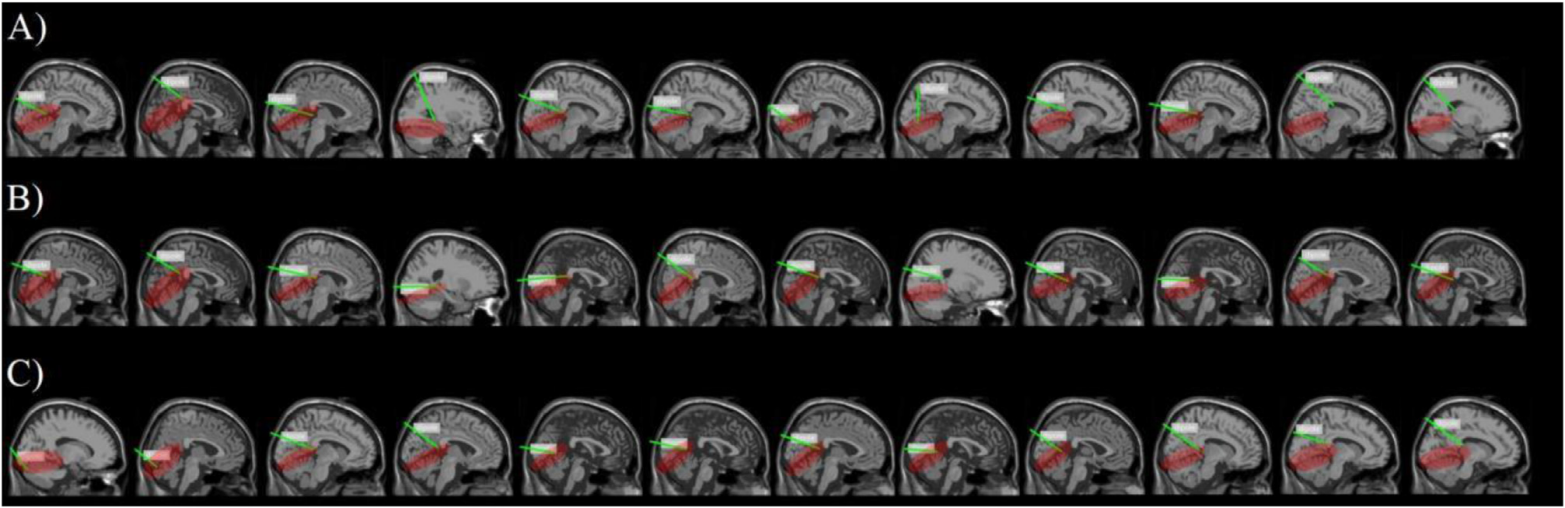
Using machine learning defined regions with source localization. Figure 15-A) The result of using machine learning to defined source location regions in team RG. Figure 15-B) The result of using machine learning to defined source location regions in team RB. Figure 15-C) The result of using machine learning to defined source location regions in team GB. 12 subjects functional anatomy, on right side view (we choose the best result of 20 subjects). Regions of visual color information were defined using machine learning decoding result. The right side view shows the extension of the DLM guided by machine learning decoding results through the V1 to endocranial region. Source localization estimates of the current DLM point to a same endocranial region (red region).

**Figure 16:**
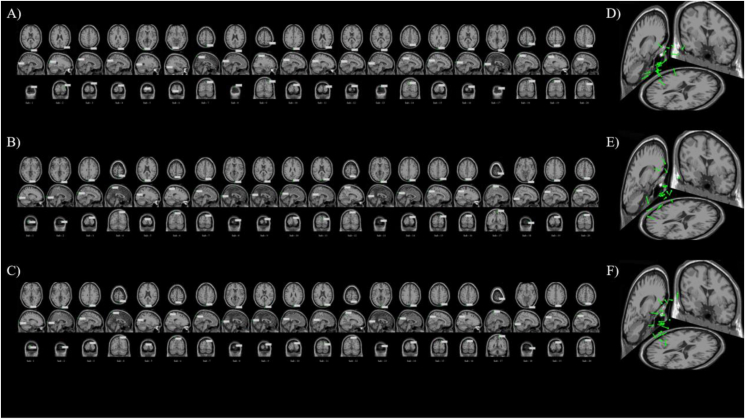
Space location reconstruction. Figure 16-A), B), C) The left inset function anatomy image from 20 subjects, on the MRI 3D-plot (right side view, rear view, and top view). Dipole location under the guided of machine learning decoding result. D E F The right inset function anatomy image from 20 subjects, reconstructing the machine learning decoding guided dipole space location.

Figure 15-A), Figure 15-B), and Figure 15-C) shows the results of machine learning guided EEG space source localization and reconstruction. According to the results of eLORETA’s traceability locating in the MRI power heat map, we use DLM to trace the source in the space location, and convert the MRI two-dimensional heat map generated by eLORETA into a three-dimensional image. The space locating of DLM can better display and help understanding the origin point that forms in the brain after nerve cells are activated. It needs to be pointed out here that it is still the machine learning decoding results guide the DLM space localization and reconstruction. Figure 16-A), Figure 16-B), and Figure 16-C) shows the results of DLM transforming the eLORETA source localization into stereo space. All 20 subjects in three teams of RG, RB, and GB received color visual stimulation. The location of the nerve cell activation source appeared in the V1. In state of active, most dipoles showed obvious directivity, but few were not very satisfactory. We further analyzed the results of satisfactory locating. By extending the vector line of the dipole pointing to the endocranial area, we found that the extension line of the retrospective positioning of the vast majority of subjects finally pointed to the calcarine sulcus (Figure 15-A, B, C). Figure 15-A), Figure 15-B), and Figure 15-C) shows the pointing results of the extension lines. The red area is the endocranial calcarine sulcus, and the terminal end of almost all extension lines (green lines) point to this area. Figure 16-D), Figure 16-E), and Figure 16-F) shows the reconstruction results of the DLM in 3D volume for 20 subjects. We merged all the dipoles of each team of 20 subjects and displayed them in the same three-dimensional space. In line with the results of the left panel and the analysis in Figure 15-A), Figure 15-B), and Figure 15-C), all the dipoles were concentrated in the V1 and most dipoles were situated in V1. There is a consistent directionality, and the final target of the dipole with the same directionality is the endocranial calcarine sulcus.

We can conclude that when the visual is stimulated by red, green, and blue, a high classification accuracy is obtained on the Pz, POz, and Oz channel of the V1 by machine learning decoding. The underlying representation of nerve cells is the endocranial calcarine sulcus. Now let us use the forward problem to explain the electrophysiological process of color stimulation-induced EEG: when the visual receives color stimulation, the nerve cells in the endocranial calcarine sulcus are activated and release a large amount of power to transmit to the V1 through skull and cortical area showed decoding features in the three channel locations of Pz, POz, and Oz.

## 4 Discussion

This study was aim to test whether EEG evoked through visual color sensing could be decoded and localized and reconstructed by machine learning. In analogy with the lower temporal resolution of fMRI and invasive approach to probing endocranial neuronal source, we obtained machine learning decoded EEG by activating brief non-invasive brain stimulation and simultaneously through cerebral cortex locating endocranial signal sources to become a possibility. Our results revealed that machine learning is not just decoding but can use for locating. To search visual color sensing neural activity location, we conducted a series of experiments. These task-irrelevant fast random stimuli revealed that color also elicits visual sensing and visual cortical excitability, as proven by the frequency evoked SSVEP. We also supplied evidence that VEP activation EEG in line with the theoretical mechanism of ERP, P300. It’s worth specifying that conducting these experiments does support and enhancement of human-computer interaction environments in a multi-dimensional manner characterized by color. They revealed the specificity of neuronal activation in the V1 by decoding differences in space channel distributions in anatomical EEGs using machine learning. They confirmed that the location of the array-like channels decoded by machine learning was in line with the location of the power distribution of VEP activation and firmly link the V1 channels to visual color sensing function. Finally, they reverse-modeled the endocranial location of the neuronal sources of these results and showed that there are indeed brain functional areas for color sensing in the brain. Overall, Our three experiments results then offer support for the exploration of endocranial neural sources through EEG inversion problems in a non-invasive setting. Reconstruction of activation locations of endocranial neural sources from machine learning decoding of visual cortex activity and function.

The phase of visual interaction reflects the nerve cells excitatory state at any moment which given of stimulation. Thus, it seems brain appears to be designed to prioritize task-relevant stimuli to generate EEG in sensorial regions, such as the visual cortex [22]. There is evidence shows that the attention can biased towards task-relevant designed to inhibit the irrelevant information processing [23] or majorization objective discriminability [24, 25] if prompted to allow time prediction of upcoming stimuli (see also speech processing studies [26] and rhythm predictions [27, 28, 29]). However, the direct-testing sensory area for this task-relevant stimulus design is still missing [30]. We found that brief color activations lead to transient sensing-related responses of neural activity and excitability in the visual cortex (Pz, POz, Oz), showing that the distribution of spatial features in V1 is aligned with the sagittal line.

The space location of visual color information processing was specific since stimulation was limited to the measures in both neural and physiological. This results is consistent with previous findings showing that the brain’s processing of color information is primarily associated with V1 areas and large-scale fronto-occipital networks [31, 32, 33, 34, 35, 36, 37, 38, 39]. In our experiments, we did not find that the decoding results of machine learning in other cortex sand out. This contrasts with previous studies of hue and luminance on the time course [40]. Common EEG decoding research directs attention to the ability of classification decoders. While choosing to limit the analysis to decoder performance in these studies, direct comparisons with our results cannot be made, difference in the design of experimental, containing the need for task-relevant designs arrangement in previous but not our work, might explain some discrepancies in the results. In our experiments, the lack of absolute analysis of good or bad decoding results in our study may not be the case. We noticed large variability in decoding outcomes across channels, we draw a tentative conclusion that the brain’s role in perceptual: The processing of the obtained color information may be located in a certain location in the V1. This does not exclude that the results of machine learning decoding in space location will have some correlation with other analysis results, and exploring the decoding effect of other better classification decoders or neural networks here may also be kick in.

In nerve cells, specific fluctuations have already revealed by several behavioral studies that are evident immediately after the presentation of visual stimuli, sudden-onset sensory events [41, 42, 43, 44]. Some of these studies also showed that this behavior rhythm sense matches the simultaneously recorded EEG period [43, 44]. These specific fluctuations in neuronal cells are likely induced by the external event of ERP, followed by regular exploration of the visual scene [42]. Here, we exploit completely different designs than the above studies, which used ERP to simulate firing of ‘neurogenic’ signals by directly activating nerve cells, rather than eliciting active execution of task-relevant through presenting a sensory events. Contrary to our findings, these preceding studies have offered evidence that perceptually informed decision-making is sampled at slower frequencies in the Cz central cerebral cortex. This discrepancy can be attributed to differences in task requirements, given that our design did not support the design of the tasks, encouraging the performance of single or multiple tasks will lead to contamination of the acquired EEG sensory information. Our design explored the specific color stimulus system (absolute pure color space), whereas the above studies using task-relevant designs may involve additional joint decoding systems.

Following the activation of the neurons, we found the differential effect of inter-channel decoding was anatomically specific, i.e. restricted to V1. Although we found quantitative results at cortical locations using the decoded results from machine learning, the traceback localization of eLORETA and DLM is still weak, so it can only be projected in 3D-MRI space by a rough transcranial simulation through the extension of vector lines to endocranial area.

In conclusion, by showing that the activity of neural activation within the V1 and the results of machine learning decoding of discriminative color information sensing align with the phase of the VEP power distribution, we provide direct evidence that machine learning can decode visual color EEG and guide endocranial traceability positioning.

## 5 Conclusion

Previous scientific studies have explored the feature of visual EEG, such as ERP theory, P300 theory, and SSVEP based on frequency features. These findings were based on joint decoding results obtained from EEG [45]. However, there is still no clear conclusion on the formation and decoding of visual color sensing that most evokes complex human senses [46]. Therefore, it is important to investigate the mechanisms by which these perceptual information are generated and the location of endocranial nerve sources. The current study specifically examined the EEG responses of optic nerve cells to color stimuli. The responses of these EEG were further explored in color vision mechanisms and neurogenesis. According to the results of this experimental, the conclusions can be as follow: (1) The EEG response of the brain’s sensing of color information exists in the V1; (2) Through machine learning, the channels containing visual color information can be forward decoded from a clear temporal resolution in the V1 Pz, POz, and Oz superior. (3) Guided by the machine learning decoding results, the position of the nerve source generated by the visual color mechanism can be traced to the vicinity of the endocranial calcarine sulcus through the EEG inverse problem.

Compared with previous studies, the innovation of this work is mainly summarized as follows: Chaudhary, M. et al. mainly focused on using different classifiers to improve the decoding efficiency, still relying on traditional spectral power features in the neural feature extraction process [47]. The approach based on cognitive tasks using color cues in developing techniques in the design of experimental paradigms. This is consistent with the traditional task-relevant design. In contrast to this study, our work proposes an independent decoding based on task-irrelevant design, which aims to exclude noise from the visual color EEG signal during decoding at its source. In addition, the traditional joint decoding requires tedious stripping and extraction in the post-processing of the collected signals, which requires certain screening of the EEG signals, during which useful in formation is easily removed. In neural feature extraction, our research mainly relies on color information EEG as a feature for classification, and gets rid of other EEG information interference that may appear in the process of traditional energy spectrum analysis. Che, X. et al. Used the recognition of color object and the memory of visual working to comprehension the visual cognitive process mechanism and assess the ability of memory [48]. The study also used a combination of memory tasks and visual stimuli for joint decoding. Although better decoding results were obtained, the results were obtained based on the subjective memory task of the brain after visual color stimulation, rather than directly evoked by sensing. In contrast to this research, our work highlights the results of emphasizing the participation of only color information without any aids and subjective judgments during the stimulus interaction phase. The purpose of this design is to retain only the brain sensing information induced by visual color stimulation in the obtained EEG signal. Torres-García, A.A. and Molinas, M. et al. analyzed the aspects of color imagine and exposure in BCIs control potential use [49]. This research adopts the same paradigm of visual interaction with the color of the red, the green, and the blue as stimulus aims as in our study. In the paradigmatic design, they used a combination of passive stimulation and motor imagery. The obtained results suggest that using both tasks together would help to effectively identify imagined and illustrated colors to control BCI. Compared with our work, this research still adopts the method of task-relevant design and joint decoding. Our work uses active, fast, and random stimuli compared to this research, a design that allows a good grasp of the overall controllability of the experiment and study compared to passive stimuli, and highlights the naturalness of the results. We have also used memory, decision-making, and judgement to compare joint decoding with independent decoding in previous studies, and have found the direction of the flow of these information in different functional regions of the brain [50]. Although the decoding effect obtained is also good, it is difficult to make a very accurate judgment in the separation of EEG signals and the authenticity of EEG information. Therefore, in this work, in order to exclude theses problems, we redesigned the experimental paradigm and innovatively used the method of task-irrelevant and independent decoding to decode visual color EEG.

In the current study, the classification decoder used SVM and FNN. However, different classifiers have a lot of performance, therefore, the problem of insufficient decoding effect and not very satisfactory classification results is revealed from the current study. To reveal the color decoding properties more important, further research is necessary, using classifiers with different performances and extending the research to dynamic colors. However, this has the potential to provide more accurate reconstructions of color EEG neural source locations for research.

## Declarations

### Author contribution statement

Yijia WU: Conceived and designed the experiments; Performed the experiments; Analyzed and interpreted the data; Contributed reagents, materials, analysis tools or data; Wrote the paper.

Yanjing Mao: Performed the experiments.

Kaiqiang Feng: Performed the experiments; Contributed reagents, materials, analysis tools or data.

Yanni Zhang, Donglai Wei, Liang Song: contributed reagents, materials, analysis tools or data.

### Funding statement

This work was supported by the 10.13039/501100012166National Key Research and Development Program of China (2018YFC0831102), the 10.13039/501100003399Science and Technology Commission of Shanghai Municipality (21JC1405300) and by the Academy for engineering & technology of 10.13039/501100003347Fudan University, and the Shanghai Key Research Laboratory of INSAI and Shanghai east-bund institute on networking systems of AI.

### Data availability statement

Data associated with this study has been deposited at https://www.scidb.cn/detail?dataSetId=5f63941f656c4a5b862bd1bc2896d3f6.

### Declaration of interest’s statement

The authors declare no conflict of interest.

### Additional information

No additional information is available for this paper.
